# Severe toxicity-free survival following acute lymphoblastic leukemia in patients aged 1–45 years: a Danish cohort study

**DOI:** 10.1038/s41375-026-02873-x

**Published:** 2026-02-10

**Authors:** Camilla Grud Nielsen, Bodil Als-Nielsen, Birgitte Klug Albertsen, Ólafur Birgir Davídsson, Andreja Dimitrijevic, Henrik Hjalgrim, Marianne Ifversen, Louise Lundgren, Line Stensig Lynggaard, Marianne Olsen, Ulrik Malthe Overgaard, Cecilie Utke Rank, Mathias Rathe, Klaus Rostgaard, Kjeld Schmiegelow, Liv Andrés-Jensen

**Affiliations:** 1 Department of Pediatrics and Adolescent Medicine, Copenhagen University Hospital, Copenhagen, Denmark; 2 Hematology, Danish Cancer Institute, Danish Cancer Society, Copenhagen, Denmark; 3 Department of Child and Adolescent Medicine, Aarhus University Hospital, Aarhus, Denmark; 4 Department of Hematology, Odense University Hospital, Odense, Denmark; 5 Department of Clinical Medicine, Copenhagen University, Copenhagen, Denmark; 6 Department of Hematology, Copenhagen University Hospital, Rigshospitalet, Copenhagen, Denmark; 7 Department of Hematology, Aarhus University Hospital, Aarhus, Denmark; 8 Network for Adolescents and Young Adults Cancer Research in Denmark (NAYAcare DK), Danish Comprehensive Cancer Center (DCCC), Aarhus, Denmark; 9 Pediatric Department, Aalborg University Hospital, Aalborg, Denmark; 10 Department of Pediatric Hematology/Oncology, Hans Christian Andersen’s Children’s Hospital, Odense University Hospital, Odense, Denmark; 11 Department of Clinical Research, University of Southern Denmark, Odense, Denmark

**Keywords:** Acute lymphocytic leukaemia, Diseases

## Abstract

With increasing survival in acute lymphoblastic leukemia (ALL), long-term toxicities have become a critical aspect. A novel measure, designated Severe Toxicity-free survival (STFS), was developed through international consensus to integrate the most severe, symptomatic organ toxicities in outcome evaluation. This measure has not been applied to real-world data before. We assessed the incidence of 21 predefined Severe Toxicities in a nationwide cohort of 506 ALL patients aged 1–45 years treated according to the NOPHO ALL2008 protocol. At five years, event-free survival was 84.4% (95% CI: 81.3–87.7%) and Severe Toxicity-event-free survival was 78.4% (95% CI: 74.9–82.1%), with significantly lower values in adults (aged 18–45 years) than children (61.6% [52.6–72.2%] vs 82.4% [78.8–86.2%]; log-rank *p* < 0.001). The most common Severe Toxicities were severe osteonecrosis limiting activities of daily function (*N* = 20) and disabling paralytic and neuropathic conditions (*N* = 16). Exploratory analyses showed that 10–17-year-olds had the highest risk of Severe Toxicities similar to that of adults. These findings highlight a burden of severe, long-term toxicities in ALL survivors overlooked by traditional outcome measures, also following frontline therapy only. STFS should be incorporated in future trials for meaningful outcome evaluation and international comparisons across treatment strategies.

## Introduction

While treatment of patients with acute lymphoblastic leukemia (ALL) has improved significantly during the past decades, the burden of long-term, treatment-related toxicities remains high [1–4]. Childhood ALL now has a particularly favorable prognosis with cure rates exceeding 90% with the best contemporary treatment regimens [5–8]. Historically, adult ALL patients have had a dismal prognosis; however, adapting pediatric treatment strategies for young adults (aged 18–45 years) has proven to be feasible and has raised five-year overall survival rates to more than 75% [9, 10].

With improved survival, there has been growing attention to long-term treatment-related toxicity [11–13]. Consequently, current treatment protocols aim not only to maximize survival but also to minimize toxicity. Nevertheless, until recently, there have been no standardized or systematic approaches to define, capture, or report even the most severe long-term toxicities. This hinders a uniform integration of toxicity into treatment outcome evaluation and, thus, meaningful comparison of both cure rates and the long-term burden of curative treatment across protocols.

Addressing this, an international collaboration of ALL experts recently established physician-derived consensus definitions of 21 severe health conditions, referred to as Severe Toxicities (ST) [14, 15]. These ST constitute the most serious treatment-related toxicities, which may be considered an unacceptable price for cure, causing significantly impaired function or increased risk of mortality. Examples include brain damage, persistent organ failure, and severely reduced physical ability in relation to osteonecrosis or paralytic, neuropathic, and movement disorders. Importantly, ST are required to be symptomatic (i.e., not require screening), objectively verifiable, and represent severe health issues that may be related to or attributable to antileukemic treatment. These consensus definitions enable the use of Severe Toxicity-free survival (STFS) as a complementary outcome, providing a more nuanced understanding of treatment outcomes beyond survival and traditional events (i.e., resistant disease, death before or in remission, relapse, and second malignant neoplasm [SMN]).

This is the first population-based cohort study to evaluate STFS. We applied the STFS measure to a Danish, nationwide cohort of 506 ALL patients aged 1–45 years, treated according to the same contemporary minimal residual disease-guided protocol. The aim of the study was to assess the occurrence of ST in both childhood and adult ALL patients and to explore associated risk factors, thereby supporting integration of toxicity outcomes into future treatment evaluation as a new standard.

## Methods

### Study design and population

We compiled a nationwide retrospective cohort of patients aged 1–45 years at the time of diagnosis of Philadelphia chromosome-negative (Ph-) ALL. We included all patients treated in Denmark between 2008 and 2019 according to the Nordic Society of Pediatric Hematology and Oncology ALL2008 (NOPHO ALL2008) protocol. All patients were diagnosed with ALL more than five years before study inclusion [16, 17]. The cohort was identified through the NOPHO ALL2008 database and consisted of 407 children and 99 adults, aged 1–17 years and 18–45 years, respectively, at the time of ALL diagnosis. There were no systematic differences between the Danish cohort and the NOPHO ALL2008 study cohort in terms of clinical characteristics, allocation, and outcome [16].

The study was registered at ClinicalTrials.gov (Identifier: NCT05639673).

### Treatment and follow-up care

The NOPHO ALL2008 protocol has been published in detail [16]. Based on biological characteristics at diagnosis and minimal residual disease in bone marrow after a 3-drug induction therapy (vincristine, doxorubicin, glucocorticosteroids, intrathecal methotrexate) on days 15 and 29, and after consolidation therapy on day 79 (or after the first two high-risk chemotherapy blocks), patients were assigned to standard risk (SR), intermediate risk (IR), high risk (HR), or HR therapy with hematopoietic stem cell transplantation (hSCT) indicated by poor response at minimal residual disease time points day 29 (≥5%) or day 79 or post 2nd high risk block (≥0.1%). Except for a difference in the capping dose of vincristine and an option for adult patients to be offered hSCT in case of *KMT2A*-rearrangements, the treatment within each risk group was independent of patient age. Patients with SR and IR ALL received conventional post-induction ALL therapy with one or two delayed intensifications, respectively, whereas patients with HR ALL received seven to nine intensive chemotherapy blocks in addition to delayed intensification and maintenance therapy. No patients received irradiation outside the hSCT setting. Oral mercaptopurine/methotrexate maintenance therapy was continued for all non-transplant patients until 2.5 years from diagnosis.

After completion of therapy, patients attended regularly scheduled follow-up visits until 60 months after the end of therapy. Beyond this time point, patients were either discharged from follow-up or continued follow-up on an individualized schedule, based on clinical needs.

### Data collection

Demographic and ALL-specific data (e.g., phenotype and treatment exposure) were obtained from the NOPHO ALL2008 database. The occurrence of 21 predefined ST was assessed by electronic medical chart review, through a combination of treatment and follow-up summaries and pre-defined, systematic keyword searches [15]. Chart review was conducted by one dedicated clinician per department (for two departments, two external reviewers performed the review). Relevant preexisting and predisposing conditions were also evaluated. If a patient fulfilled the criteria for a specific ST prior to their leukemia diagnosis, the condition was not classified as an ST event. Such patients remained eligible for inclusion in analyses of overall ST occurrence (e.g., STFS or cumulative incidence of ST), as they were still at risk for other types of ST during follow-up.

For patients aged <18 years at the time of diagnosis, data were retrieved from the period between ALL diagnosis and November 1st, 2023. For patients aged ≥18 years at the time of diagnosis, data were retrieved from the period between ALL diagnosis and September 30th, 2024 (the difference in end dates reflects administrative differences in data access approvals). Data were collected using Research Electronic Data Capture (REDCap) hosted at Copenhagen University Hospital.

### Severe toxicities (ST) definitions

The definitions of ST used in this study are based on a revised version of the original consensus definitions [14, 15]. As specified in the modified definitions, ST could occur at any point after the initiation of treatment. A 12-month persistence requirement was applied to conditions with potentially fluctuating and reversible courses, e.g., heart failure and neuropathy. For cognitive dysfunction and psychiatric disorders, the condition was required to persist for ≥12 months *after* the completion of antileukemic treatment, to avoid the potential impact of other treatment-related side effects. Specifically, a two-tiered grading system for cognitive dysfunction was introduced, categorizing it as “possible” or “verified”. Only “verified” cases were included in the analyses, acknowledging inherent variability in assessment and documentation.

Further details regarding definitions and the classification process are available in the Supplementary (sections 1 and 2).

### Data analysis

The occurrence of ST was assessed using time-to-event analyses. Time was measured in years since diagnosis. Patients were censored at the last day of follow-up if no events had occurred. The evaluated outcomes included: (i) Overall survival (OS): Time from diagnosis to death from any cause; (ii) Event-free survival (EFS): Time from diagnosis to the first occurrence of resistant disease, relapse, second malignant neoplasm (SMN), or death; (iii) Severe Toxicity-free survival (STFS): Time from diagnosis to the first occurrence of ST or death; iv) Severe Toxicity-event-free survival (ST-EFS): Time from diagnosis to the first occurrence of ST, resistant disease, relapse, SMN, or death.

Patients who died from an ST, e.g., organ failure, before fulfilling the 12-month criterion were censored at the time of death. Notably, SMN was included in both the traditional EFS definition and as an ST, thereby contributing to both EFS and STFS. For transparency, STFS estimates are presented both with and without SMN.

The timing of an ST event was defined as the date when the relevant criteria were fulfilled, which, for several definitions, required a duration of at least 12 months.

Kaplan-Meier estimates were used to estimate the probability of remaining event-free over time. Differences in survival curves between children and adults were compared using the log-rank test. The cumulative incidence of ST was estimated using the Aalen-Johansen method, accounting for death as a competing risk. When analyzing specific ST, other ST did not constitute censoring events (e.g., osteonecrosis did not end follow-up when assessing cumulative incidence for SMN).

Exploratory risk factor analyses were also conducted. We fitted a Cox proportional hazards model, adjusting for sex, age group, and relapse status (as a time-dependent covariate), and stratified by risk group to account for differences in baseline hazards. The isolated impact of hSCT in first clinical remission (CR1) was investigated in a separate Cox model in which patients were censored at the time of relapse. Model assumptions were evaluated using Schoenfeld residuals. Associations are presented with nominal 95% confidence intervals. No adjustments were made for multiple testing.

We generated adjusted survival curves from a simplified Cox model to visualize the association between age and risk of ST. Details are provided in the Supplementary (section 5).

### Ethics approval and consent to participate

All methods were performed in accordance with relevant guidelines and regulations. The study was registered in the Capital Region of Denmark’s internal research register (Privacy, P-2022-622, P-2024-17486) and approved for access to medical records by the Capital Region’s patient record team (R-22029073, R-24067893) and all participating departments; the requirement for informed consent was waived by the Capital Region’s patient record team in accordance with Danish legislation.

## Results

### Cohort characteristics

Among 407 children and 99 adults, the median follow-up time was 7.9 years for children and 6.0 years for adults (Table 1). Relapse occurred in 8.1% of pediatric patients (5-year cumulative incidence [CIR], 7.0%; 95% CI: 4.7–9.7%) and 19.2% of adult patients (5-year CIR, 16.8%; 95% CI, 10.0–25.0%). hSCT was performed in 12% of children and 36.4% of adults. The characteristics of the study cohort are presented in Table 1.

**Table 1:** Patient Characteristics.

	1-17 years	18-45 years	Overall
	(*N* = 407)	(*N* = 99)	(*N* = 506)
**Sex**			
Male	226 (55.5%)	60 (60.6%)	286 (56.5%)
Female	181 (44.5%)	39 (39.4%)	220 (43.5%)
**Phenotype**			
B-lineage	340 (83.5%)	65 (65.7%)	405 (80.0%)
T-lineage	62 (15.2%)	33 (33.3%)	95 (18.8%)
MPAL	4 (1.0%)	1 (1.0%)	5 (1.0%)
Other*	1 (0.2%)	0 (0%)	1 (0.2%)
Risk group distribution**			
SR	165 (40.5%)	16 (16.2%)	181 (35.8%)
IR	164 (40.3%)	44 (44.4%)	208 (41.1%)
HR	77 (18.9%)	39 (39.4%)	116 (22.9%)
**hSCT**			
No	358 (88.0%)	63 (63.6%)	421 (83.2%)
Yes (CR1)	31 (7.6%)	25 (25.3)	56 (11.1%)
Yes (CR2 or later)	18 (4.4%)	11 (11.1%)	29 (5.7%)
**Relapse**			
No	374 (91.9%)	80 (80.8%)	454 (89.7%)
Yes	33 (8.1%)	19 (19.2%)	52 (10.3%)
**Follow-up time (years)**			
Mean (SD)	8.07 (3.27)	6.32 (3.38)	7.73 (3.36)
Median [Min, Max]	7.90 [0, 14.8]	6.00 [0.10, 16.2]	7.70 [0, 16.2]

*Other: blastic plasmacytoid dendritic cell leukemia.

**One patient died before being assigned to a risk group. This patient was excluded from analyses, including risk groups, but included in the remaining analyses.

*MPAL*, mixed phenotype acute leukemia, *SR* standard risk, *IR* intermediate risk, *HR* high risk, *hSCT* hematopoietic stem cell transplantation.

### Occurrence of ST

Among the 407 children, 36 (8.8%) were classified with at least one ST, nine of whom had a relapse prior to the ST. Among the 99 adult patients, 16 (16.2%) were classified with at least one ST, none of whom had experienced relapse prior to the ST. Notably, 13 of 19 adult patients with a relapse died within the study timeframe, eight within 12 months after relapse. Among the 52 patients who experienced at least one ST, 46 were alive at the time of last follow-up, with a median follow-up time of 4.6 years since ST occurrence. A total of 43 patients had a single ST, seven were classified with two ST (osteonecrosis being one of them in six patients), and two patients experienced more than two ST. The most common ST was osteonecrosis, affecting 20 patients. Of these, seven (35%) had one joint affected, seven (35%) had two joints affected, and six (30%) had involvement of three or more joints.

Table 2 shows all the ST observed in the cohort. Brief descriptions of cases are available in the Supplementary Material (sections 3 and 4).

**Table 2:** Severe toxicities (ST) observed in the cohort.

Severe Toxicity (ST)	Number of patients	Median time to fulfilled ST criteria (years, 25–75% IQR)	Relapse and/or HSCT prior to ST
Osteonecrosis	20	3.0 (2.4–3.8)	5/20
PNMM	16	1.4 (1.2–2.3)	10/16
Diabetes (IDDM)	7	1.2 (1.0–4.3)	2/7
SMN	7	4.6 (3.3–6.4)	3/7
Psychiatric disease	4	5.3 (5.1–6.5)	2/4
Gastrointestinal failure	3	1.5 (1.3–1.6)	2/3
Cognitive dysfunction	2	2.7 (2.2–3.1)	1/2
Seizures	2	2.7 (2.0–3.4)	1/2
Blind	1	1.1 (*NA*)	1/1
Heart valve dysfunction	1	7.6 (*NA*)	0/1
Hepatic failure	1	6.3 (*NA*)	1/1
Amputation and physical deformation	1	0.2 (*NA*)	0/1

The table lists the number of patients affected by each ST, median time to fulfilling the ST criteria (in years, with IQR), and the proportion with prior relapse and/or hSCT. The following STs were not observed in this cohort: hearing loss, heart failure, coronary artery disease, arrhythmia, renal failure, pulmonary failure, vocal cord paralysis, cytopenia, and immunodeficiency.

*IQR* interquartile range, *hSCT* hematopoietic stem cell transplantation, *PNMM* paralytic, neuropathic, myopathic, and movement disorders, *IDDM* insulin-dependent diabetes mellitus, *SMN* second malignant neoplasms and benign central nervous system tumors.

### Severe Toxicity-free survival (STFS)

**Fig. 1:**
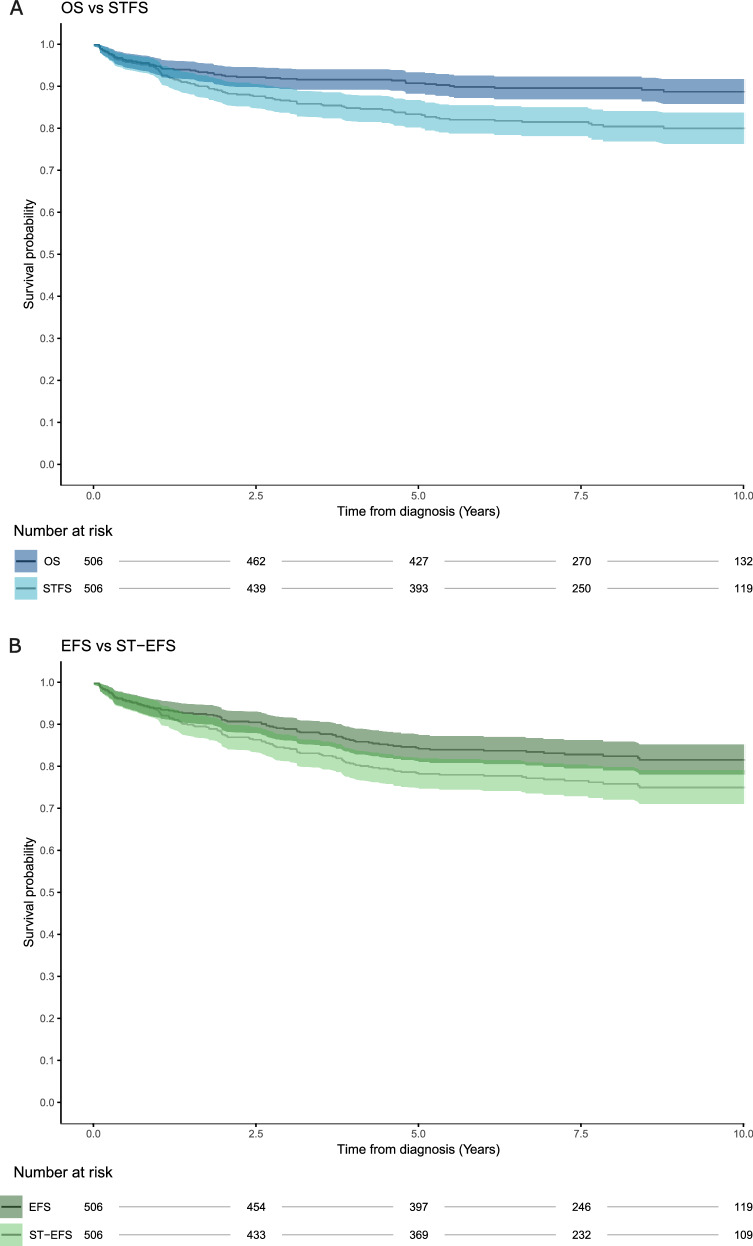
Overall survival (OS), severe toxicity-free survival (STFS), event free survival (EFS), and severe toxicity-event free survival (ST-EFS) in patients with ALL aged 1–45 years. Kaplan-Meier curves showing **A** overall OS and STFS; **B** EFS and ST-EFS in children and adults. STFS and ST-EFS include Severe Toxicities as events. Patients without events were censored at the last follow-up. Note that second malignant neoplasms (SMN) are included in both the EFS and STFS definitions.

Five-year OS was high in both children (92.4%) and adults (83.5%), whereas STFS was lower (86.7% and 69.0%) (Table 3). These differences in OS and STFS between children and adults were statistically significant (log-rank *p* < 0.001). Excluding SMNs had only a minor impact on STFS estimates. Kaplan-Meier curves for the full cohort are shown in Fig. 1; corresponding five-year estimates are provided in Table 3.

**Table 3:** Five-year survival estimates.

Outcome	Children (95% CI)	Adults (95% CI)	Total (95% CI)
OS	92.4% (89.8–95.0)	83.5% (76.4–91.2)	90.7% (88.1–93.2%)
EFS	86.9% (83.7–90.3)	74.0% (65.7–83.4)	84.4% (81.3–87.7%)
STFS	86.7% (83.4–90.1)	69.0% (60.4–78.9)	83.3% (80.1–86.6%)
STFS (SMN excluded)	87.2% (84.0–90.5)	70.1% (61.6–79.9)	83.9% (80.7–87.2%)
ST-EFS	82.4% (78.8–86.2)	61.6% (52.6–72.2)	78.4% (74.9–82.1%)

*OS* overall survival, *EFS* event-free survival, *STFS* severe toxicity-free survival, *ST-EFS* severe toxicity-event-free survival, *SMN* second malignant neoplasms and benign central nervous system tumors.

### Cumulative incidence of ST

**Fig. 2:**
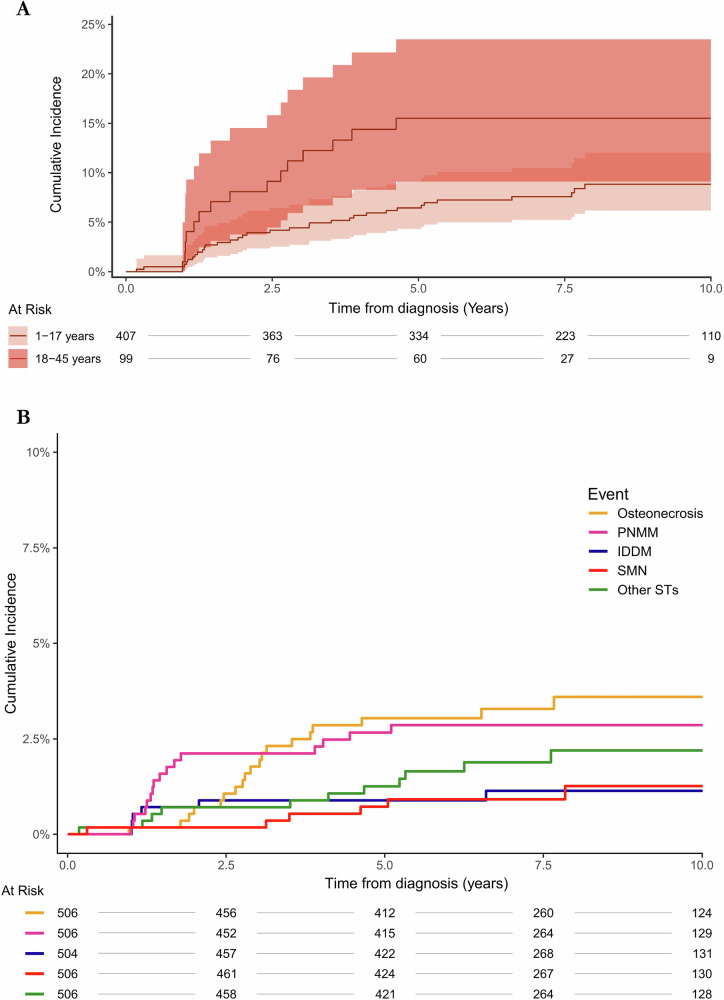
Cumulative incidence of Severe Toxicities in children and adults with ALL. **A** The cumulative incidence of Severe Toxicities in children and adults, treating death as a competing risk. **B** The cumulative incidence of the most frequently observed Severe Toxicities (ST) in both children and adults, treating death as a competing risk. “Other” ST include psychiatric disease, gastrointestinal failure, cognitive dysfunction, seizures (drug-resistant epilepsy or requiring neurosurgery), heart valve dysfunction, hepatic failure, and amputation and physical deformation. IDDM insulin-dependent diabetes, PNMM paralytic, neuropathic, myopathic, and movement disorders, SMN second malignant neoplasm and benign central nervous system tumors.

The cumulative incidence of any ST was higher in adults compared to children (Fig. 2A). At five years, the cumulative incidence in adults was 15.5% (95% CI: 9.1–23.4%) compared to 6.4% (95% CI: 4.3–9.1%) in children. Beyond five years, ST were observed in children, including SMN, IDDM, osteonecrosis, paralytic, neuropathic, myopathic, and movement disorders, heart valve dysfunction, and psychiatric disease.

The cumulative incidence by risk group was explored in unadjusted supplementary analyses (Supplementary Fig. 1), which indicated higher incidence rates in the IR and HR groups compared to the SR group. It should be noted that age has a strong impact on primary risk stratifying criteria, immunophenotype, and minimal residual disease, with a higher proportion of older patients being assigned to more advanced risk groups (Supplementary Table 5) [17].

In additional analyses, censoring at relapse to evaluate first-line therapy only, the cumulative incidence of ST in children decreased to 5.8% (95% CI: 3.8–8.5%) at 5 years. The corresponding estimate for adults increased slightly from 15.5% to 16.9% 95% CI: (9.9–25.6%), reflecting that only 8 of the 19 adult patients with relapse achieved 2-year survival after relapse, and of these, none experienced ST after relapse (Supplementary Fig. 2).

The cumulative incidence of the most common ST (osteonecrosis (*n* = 20), paralytic, neuropathic, myopathic and other movement disorders (*n* = 16), IDDM (*n* = 7; 3 after pancreatitis), and SMN (*n* = 7) is shown along with “Other ST” in Fig. 2B.

### Risk factors for developing ST

To explore age-related differences, the pediatric population was stratified into narrower age intervals. The cumulative incidence was lowest among the youngest children (1–4 years), at 1.5% (95% CI: 0.4–4.0%) at 5 years, while older age groups showed higher estimates (Supplementary Fig. 3).

To further explore the role of age, we generated adjusted survival curves stratified by risk group. These analyses demonstrated a higher risk of ST with increasing age across risk groups (Supplementary Fig. 4).

In a Cox model stratified by risk group, older children, adolescents, and adults all had an increased risk of developing ST compared to the youngest group (1–4 years), with HRs of 4.0 (95% CI: 1.4–11.3) for 5–9-year-olds, 8.6 (95% CI: 3.1–24.0) for 10–17-year-olds and 6.6 (95% CI: 2.3–18.7) for adults (18–45 years). Relapse, and consequently second-line therapies including hSCT, were strongly associated with ST (HR 6.4; 95% CI: 2.9–14.4). Sex was not independently associated with an increased risk of ST (Supplementary Fig. 5).

hSCT in CR1 was associated with an increased risk of ST (HR 2.3; 95% CI: 0.9–5.9), although not statistically significant (Supplementary Table 11). Among HR patients, ST risk did not differ significantly between those receiving hSCT in CR1 and those treated with chemotherapy alone (Supplementary Table 12).

## Discussion

As cure rates for many cancers continue to rise, particularly for pediatric patients, the long-term burden of severe, treatment-related toxicities becomes an increasingly critical issue. ALL exemplifies this challenge with current survival rates above 90%, highlighting the limitations of traditional treatment evaluation that overlook the long-term consequences of therapy. We observe that approximately 10% of ALL patients experience at least one ST, reflecting a substantial burden not captured by conventional outcomes such as OS or EFS. While 5-year OS remains high, 92.4% in children and 83.5% in adults, the corresponding STFS probabilities were markedly lower at 86.7% and 69.0%, respectively. This discrepancy highlights how traditional survival endpoints may overestimate treatment success by neglecting the overall burden of even the most serious long-term consequences of treatment. As newer, immunotherapy-based strategies begin to redefine antileukemic treatment [18–20], a systematic and consensus-driven approach to capture severe, long-term toxicity is more crucial than ever.

While adults had a higher overall frequency of ST compared to children, older children and adolescents (10–17 years) appeared with a risk of ST comparable to or exceeding that of adults. In contrast, the youngest children (aged 1–4 years) had a significantly lower cumulative incidence of ST, emphasizing that simply categorizing patients as children and adults can mask important differences in the frequency of ST among younger versus older pediatric patients. Age-related susceptibility to specific toxicities, such as osteonecrosis—the most frequent ST in this study, which in this context refers only to the most severe cases requiring joint arthroplasty or causing long-lasting major functional impairment—likely contributes to this pattern [21–23]. These findings highlight a particularly vulnerable group, the adolescents and young adults, in whom treatment adjustments and preventive initiatives, such as modifying the dexamethasone schedule and managing hyperlipidemia and hypertension, could provide substantial clinical benefits [24–26].

The observed age-related differences in ST occurrence may partly reflect treatment stratification, as adolescents and young adults are more often assigned to higher-risk groups due to unfavorable immunophenotypes or cytogenetic features [9, 17, 27, 28]. Exploratory unadjusted analyses also suggested higher cumulative incidence rates of ST in IR and HR groups, possibly reflecting higher treatment intensity. Due to the sample size, it was not possible to separate the effects of age and risk groups. However, even when accounting for the risk group, ST risk increased with age. Relapse was also strongly associated with ST risk, likely reflecting both increased disease burden and intensified treatment. Although the isolated impact of hSCT in CR1 did not reach statistical significance, this is possibly influenced by the limited sample size and does not exclude a potential association. The suggested increased ST risk warrants further investigation in larger cohorts, including the impact of total body irradiation, which was not assessed in this study due to limited sample size. Notably, numerous ST occurred in patients who received only frontline therapy, underscoring that primary treatment also carries a risk of significant long-term toxicity.

Paralytic, neuropathic, myopathic, and movement disorders were the second most frequent ST and typically manifested as long-lasting peripheral neuropathy requiring assistive devices (e.g., wheelchair or walking aid), or as paralysis following central neurotoxicity or transverse myelitis. Peripheral neuropathy, a well-known vincristine-related toxicity, is generally reversible; however, it is also known to persist in some cases [21–23]. Our findings highlight a subset of patients who experience persistent and severely impaired physical function lasting 12 months or more due to these conditions. Future studies focusing on the specific diagnoses and underlying mechanisms contributing to these prolonged impairments are needed. This can also include identifying patients at increased risk, e.g., due to genetic predisposition such as *CEP72* variants [29], clarifying the impact of dose modification in cases of severe neuropathy, potentially through randomized trials, and early physical activity to preserve physical function [30].

Other frequent ST included IDDM and SMN, both potential consequences of antileukemic therapy, although genetic and metabolic predispositions may also contribute [31–35]. Additionally, individually rare conditions, such as persistent hepatic failure and blindness, also contributed to the overall burden of ST, representing 22% of ST events. Nine patients experienced more than one ST, and, in addition, among 20 patients with osteonecrosis, 65% involved two or more joints, indicating a higher disease burden than what single-event classifications capture.

Whether the occurrence of ST impacts survival outcomes is challenging to determine, partly due to the heterogeneity of the conditions. Some toxicities may lead to treatment modifications with potential effects on prognosis, for instance, asparaginase-associated pancreatitis resulting in asparaginase truncation, which has been linked to poorer outcomes [31]. Conversely, osteonecrosis has been associated with superior outcomes, possibly reflecting more intensive prior therapy or underlying host-related factors [32].

To address causality, preexisting conditions were systematically assessed and found to be rare: six patients had Down syndrome (none developed an ST), and only two others had conditions precluding ST classification. This issue is likely less problematic in pediatric populations than in older patient groups. In cases with known genetic or comorbid predispositions, e.g., Li-Fraumeni syndrome or prior psychiatric illness, we acknowledge that the development of an ST may not be solely attributable to treatment. However, treatment can contribute to their progression, as toxicity development is often multifactorial, involving a combination of patient-specific, genetic, and treatment-related factors. These factors were not adjusted for due to data limitations.

While the consensus definitions of ST provide a consistent framework for classification, certain limitations inherent to retrospective data collection should be acknowledged; follow-up intervals vary, and clinical documentation may be sparse. However, as the study included all patients treated in Denmark during the study period, and we had national approval to access all medical charts, the risk of reporting bias is considered low. The 21 ST are, by definition, severe, symptomatic conditions that are likely to lead to health care contacts and corresponding clinical documentation. Nevertheless, a potential underreporting of events cannot be excluded. For fluctuating or potentially reversible conditions, a duration of 12 months was required. This persistence criterion ensures conservative and consistent classification, but may also overestimate long-term burden, as some conditions may occasionally improve or resolve over time. This was observed in five patients with steroid-related IDDM who no longer required insulin after treatment cessation. In future registrations of ST, it should be considered to restrict the classification of IDDM to cases persisting beyond therapy, aligning with the approach used for cognitive dysfunction and psychiatric diseases. It also highlights the importance of relevant laboratory assessments (e.g., HbA1c, fasting glucose, and C-peptide) in determining whether it reflects unnecessary continuation of insulin during a treatment phase with hyperglycemia (e.g., glucocorticosteroid- and asparaginase-based reinduction; acute pancreatitis) or a true persistence of insulin-dependent diabetes. Another patient with osteonecrosis experienced marked symptom improvement during bisphosphonate treatment, illustrating that as effective treatments emerge, conditions may no longer meet the severity threshold for ST classification.

STFS and ST-EFS are derived measures that can be applied to any cancer, with two additional limitations. First, older patients may be burdened by age-dependent co-morbidities and organ dysfunctions that can be difficult to disentangle from treatment-induced ST, and which by themselves can increase the risk of other ST. Second, some ST can be an intended part of curative treatment, e.g., limb amputation in osteosarcoma. Accordingly, what ST constitutes an “unacceptable price of cure” may vary slightly depending on context. Nonetheless, while the toxicity burden may be weighed differently in high-risk or relapsed patients, systematic mapping remains valuable for understanding the overall long-term impact of treatment. Notably, STFS and ST-EFS are composite endpoints that encompass both indicators of undertreatment (relapse, resistant disease) and overtreatment (ST, treatment-related mortality), complicating the interpretation of this measure in isolation. They should therefore be considered complementary to traditional endpoints.

Nevertheless, the total health burden experienced by the survivors naturally extends beyond the occurrence of physician-defined ST. A more comprehensive understanding of the burden of therapy, including asymptomatic issues such as fertility (originally excluded as an ST due to the lack of common screening strategies and therefore lack of comparable data) and subjective conditions such as fatigue or pain, requires the inclusion of survivors’ perspectives. While important, an assessment of such issues was outside the scope of this study. Future studies should explore how survivors experience and live with both ST and other serious, therapy-related health issues that are relevant to them.

Prospective and systematic registration of ST should be routinely integrated into follow-up visits. Based on our experience in this study, such registration is both feasible and efficient; documentation for most patients can likely be completed within minutes by healthcare professionals who regularly see the patients during treatment and follow-up, as the majority do not exhibit qualifying symptoms. The need for lifelong follow-up remains uncertain. However, systematic monitoring of ST could help identify vulnerable individuals who may benefit from more intensive, personalized follow-up. The most frequent ST observed in this study should guide future research focus areas, including exploration of risk factors and potential treatment modifications aimed at preventing these toxicities, particularly in adolescents and young adults, who represent the most vulnerable population in this study, consistent with previous findings [27, 28, 33]. Ultimately, our findings support the incorporation of ST registration and the use of STFS prospectively as a clinically meaningful endpoint in future treatment evaluations. This approach provides a more comprehensive assessment of long-term treatment burden, enabling meaningful international comparisons of toxicity outcomes across treatment strategies.

## Supplementary information

Supplementary Material

## Data Availability

Individual participant data will not be publicly shared due to ethical and legal restrictions. A waiver of informed consent was given by the Capital Region’s patient record team in Denmark. Although identifiers have been removed where possible, the dataset cannot be anonymized sufficiently to prevent de-identification. Data sharing requests for specific research purposes may be considered on an individual basis and require approval from the relevant ethics committee. Study protocol and statistical analysis plan are available at ClinicalTrials.gov (Identifier: NCT05639673).

## Code availability

The analysis code is available from the authors upon reasonable request and after individual assessment by the authors. All analysis were done using R (version 4.4.2; R Foundation for Statistical Computing, Vienna, Austria).
