# Supplementary material for: Severe toxicity-free survival following acute lymphoblastic leukemia in patients aged 1–45 years: a Danish cohort study
Source: Leukemia. 2026 Feb 10;40(3):630–7. doi: 10.1038/s41375-026-02873-x (PMC12960229; doi:10.1038/s41375-026-02873-x)
Supplement: Supplementary file 1 — Supplementary Material [file 41375_2026_2873_MOESM1_ESM.pdf]

Supplementary Material

Table of content

1. Severe Toxicity definitions 2

1.1. Modified consensus definitions 2

1.1.1. Minor clarifications 4

1.1.2. Application of the 12 months duration criterion 5

2. Data collection 5

2.1. Workflow 5

2.2. Supplementary classification procedures 5

2.3. Pre-existing conditions 6

2.4. Cognitive dysfunction 8

3. Examples of Severe Toxicity cases 11

4. Resolved Severe Toxicity cases 12

5. Analyses 14

5.1. Cumulative incidence of Severe Toxicities by risk groups 14

5.1.1. Risk group distribution across age categories 14

5.1.2. Cumulative incidence of Severe Toxicities across risk groups 15

5.2. Cumulative incidence of Severe Toxicities (censoring at relapse) 16

5.3. Exploratory risk factor analyses 17

5.3.1. Cumulative incidence of Severe Toxicities by finer age groups 17

5.3.2. Adjusted survival curves 18

5.3.3. Cox proportional hazards models 20

6. References 22

1. Severe Toxicity definitions

1.1. Modified consensus definitions

Supplementary Table 1: Modified consensus definitions of the 21 Severe Toxicities (1).

Severe Toxicity (ST)	Consensus definition, time of ST and additional notes
Hearing loss	*Definition*: Persisting bilateral hearing loss emerging during or after anticancer therapy and defined as >40 dB hearing loss at ≤2 kHz that persist for ≥12 months or requiring cochlear implant.<br>*Time of ST:* Date when at least one of the criteria are met for both ears, i.e., both ears having audiometry identified >40 dB hearing loss at ≤2 kHz that has persisted for ≥12 months and/or cochlear implant surgery (whichever occurs first).
Blindness	*Definition:* Untreatable blindness emerging during or after anticancer therapy, defined as visual acuity of <20/200 or a corresponding visual field loss to <10° in the stronger eye with the best possible correction.<br>*Time of ST:* Date when blindness is identified as visual acuity of <20/200 or a corresponding visual field loss <10° in the stronger eye with the best possible correction.
Heart failure	*Definition:* Persisting (≥12 months), symptomatic cardiac dysfunction emerging during or after anticancer therapy and defined by a decrease in left ventricular ejection fraction to a value <40% or fractional shortening to <20% and one of the following: i) age 0-1 years: marked tachypnoea or diaphoresis with feeding or prolonged feeding times with growth failure or tachypnoea, retractions, grunting, or diaphoresis at rest*, ii) age 1-17.9 years: marked dyspnea on exertion or at rest*, or age ≥18 years: marked dyspnea, palpitations or anginal pain on exertion or at rest**; or symptomatic cardiac dysfunction requiring heart transplantation.<br>*Time of ST:* Date when the condition fulfilling the clinical <u>and</u> paraclinical criteria has persisted for 12 months, or when the patient is referred for transplantation (whichever occurs first).<br>*Additional notes:* Screening of all patients with echocardiographic measures is not required, but echocardiographic confirmation is required for inclusion as ST. Echocardiographic measures are provided because international surveillance guidelines accept its use as the primary surveillance tool for cardiotoxicity. A repeat echocardiogram is expected to be done at least 1 week apart to confirm cardiac dysfunction.
Coronary artery disease	*Definition:* Coronary artery disease emerging during or after anticancer therapy and resulting in myocardial infarction, or requiring angioplasty (balloon or stent), or coronary bypass surgery.<br>*Time of ST:* Date of myocardial infarction or when angioplasty (balloon or stent) or coronary bypass surgery is performed (whichever occurs first).
Arrhythmia	*Definition:* Arrhythmia emerging during or after anticancer therapy, requiring a pacemaker or an implantable cardioverter defibrillator.<br>*Time of ST:* Date when pacemaker or ICD is implanted.
Heart valve disease	*Definition:* Heart valve dysfunction emerging during or after anticancer therapy and requiring surgical valve replacement.<br>*Time of ST:* Date when surgical valve replacement is performed.
Gastrointestinal failure	*Definition:* Gastrointestinal failure emerging during or after anticancer therapy, resulting in persistently (≥12 months) requiring parenteral nutrition, or placement of a PEG tube due to physical inability to eat or swallow that persist for ≥12 months, or placement of a stoma that persist for ≥12 months.<br>*Time of ST:* Date when the condition fulfilling the clinical criteria in the ST definition has persisted for 12 months.
Hepatic failure	*Definition:* Severe and persisting (≥12 months) hepatobiliary failure emerging during or after anticancer therapy, and defined as any of the following: symptomatic, decompensated liver disease including cirrhosis and portal hypertension that is not responsive to pharmacologic and endoscopic management; or any hepatobiliary failure requiring liver transplantation.<br>*Time of ST:* Date when the condition fulfilling the clinical criteria in the ST definition has persisted for 12 months or when the patient is referred for transplantation (whichever occurs first).<br>*Additional notes:* Typical symptoms of hepatic failure include fatigue, gum bleeding, epistaxis, itching, and icterus in all age groups in addition to impaired growth and delayed puberty in children. Patients who undergo a portosystemic shunt for hepatic disease are included in this definition because shunts are usually reserved for refractory disease, which may serve as a bridge to liver transplant.

Insulin dependent diabetes	*Definition:* Persisting (≥12 months) insulin dependent diabetes emerging during or after anticancer therapy.<br>*Time of ST:* Date when the condition fulfilling the clinical criteria in the ST definition has persisted for 12 months.<br>*Additional notes:* Insulin dependent diabetes is treatable; however, is included because of substantial risk of cardiovascular disease and end-organ failure.
Renal failure	*Definition:* Persisting (≥12 months) loss of kidney function emerging during or after anticancer therapy that requires dialysis or renal transplantation.<br>*Time of ST:* Date when the condition fulfilling the clinical criteria in the ST definition has persisted for 12 months or when the patient is referred for transplantation (whichever occurs first).
Pulmonary failure	*Definition:* Chronic lung failure (including pulmonary fibrosis and bronchiolitis obliterans) emerging during or after anticancer therapy and requiring daily oxygen supplement (≥12 months) or lung transplantation.<br>*Time of ST:* Date when the condition fulfilling the clinical criteria in the ST definition has persisted for 12 months or when the patient is referred for transplantation (whichever occurs first).
Osteonecrosis	*Definition:* Osteonecrosis occurring during or after anticancer therapy and requiring total joint arthroplasty; or resulting in grade 4 toxicity according to the Ponte di Legno Toxicity Working Group Criteria (i.e., symptomatic with deformation by imaging of one or more joints or substantially affecting self-care ADL* for ≥12 months).<br>*E.g., requiring daily assistance beyond what is considered age-appropriate with at least one self-care ADL and/or requiring instrumental aid, such as wheelchair or walking stick, for mobility.<br>*Time of ST:* Date when the condition fulfilling the clinical criteria in the ST definition has persisted for 12 months or when total joint arthroplasty is performed (whichever occurs first).<br>*Additional notes:* Examples of self-care ADL include grooming/personal hygiene, dressing, toileting/continence, transferring/ambulating, and eating.
Amputation and physical deformation	*Definition:* Amputation of extremities, severe spinal deformation, and disabling scleroderma, scarring, or contractions affecting self-care ADL* substantially for ≥12 months or causing substantial facial disfigurement, and defined as follows: lower limb amputation (proximal to ankle), upper limb amputation (proximal to wrist), scoliosis, kyphosis, or lordosis affecting self-care ADL substantially, scarring or contractions affecting range of movement that affects self-care ADL substantially, scleroderma affecting self-care ADL substantially, amputation of nose, amputation of one or both eyes, complete facial palsy (unilateral- or bilateral). Conditions emerging during or after anticancer therapy are included.<br>*E.g., requiring daily assistance beyond what is considered age-appropriate with at least one self-care ADL and/or requiring instrumental aid, such as a wheelchair or walking stick, for mobility.<br>*Time of ST:* Date when amputation is performed, or when one of the following conditions fulfilling the clinical criteria in the ST definition has persisted for ≥12 months: scoliosis, kyphosis, lordosis, scarring or contractions, or scleroderma, or when diagnosed with complete facial palsy (unilateral or bilateral).<br>*Additional notes:* Examples of self-care ADL include grooming/personal hygiene, dressing, toileting/continence, transferring/ambulating, and eating.
Cognitive dysfunction	*Definition:* Any substantial impairment of neurocognitive functions* emerging during or after anticancer therapy, that affects instrumental ADL** substantially and persist for ≥12 months <u>after</u> ending anticancer therapy.<br>*E.g., executive function (planning and organization), sustained attention, memory (particularly visual sequencing, temporal memory), processing speed, visual-motor integration, fine motor dexterity, diminished performance on IQ-test, and learning deficits).<br>**E.g., severely restricted participation in school, vocational training, practice, and career, and/or requiring daily assistance beyond what is considered age-appropriate with other key activities of instrumental ADL.<br>*Time of ST:* Date when the condition fulfilling the clinical criteria in the ST definition has persisted for 12 months in the period <u>after</u> ending anticancer therapy.<br>*Additional notes:* Cognitive dysfunction emerging after ending anticancer therapy must persist for ≥12 months to be classified as an ST. Pre-existing conditions relevant for this condition include not having met normal developmental milestones and/or evidence of developmental delay at the time of diagnosis. Other examples of instrumental ADL include cooking, cleaning, managing finances, managing medications.<br><u>"Verified" cognitive dysfunction is defined as:</u> The patient lives in an institution due to cognitive dysfunction and/or the patient requires daily assistance with instrumental ADL due to cognitive dysfunction.<br><u>"Possible" cognitive dysfunction is defined as:</u> The patient scores below normal range (<2.5 percentile) in neuropsychological test[§], but the degree of impact on instrumental ADL is uncertain and/or the patient has substantially impaired instrumental ADL, but it is uncertain if it depends on this specific condition or is due to other reasons, e.g., psychiatric disease.<br>§ Not specified due to variety in instruments used across centers. Neuropsychological testing is likely to be performed in patients with severe cognitive dysfunction but is not required for inclusion in the STFS measure.

Seizures	*Definition:* Seizures emerging during or after anticancer therapy that require neurosurgical intervention to reach seizure control, or that fulfil the International League Against Epilepsy definition for drug-resistant epilepsy ("defined as failure of adequate trials of two tolerated and appropriately chosen and used anti-epileptic drug schedules (whether as monotherapies or in combination) to reach sustained seizure freedom")†.<br>*Time of ST:* Date when the patient experiences a seizure despite adequate trials of two tolerated and appropriately chosen and used anti-epileptic drug schedules, or when neurosurgery is performed to reach seizure control (whichever occurs first).
Psychiatric disease	*Definition:* Any psychiatric disorder emerging during or after anticancer therapy, that is severe enough to require mental health input (psychology or psychiatry), and affects instrumental ADL substantially* and persist for ≥12 months <u>after</u> ending anticancer therapy.<br>*E.g., severely restricted participation in school, vocational training, practice, and career, and/or requiring daily assistance beyond what is considered age-appropriate with other key activities of instrumental ADL.<br>*Time of ST:* Date when the condition fulfilling the clinical criteria in the ST definition has persisted for 12 months <u>after</u> ending anticancer therapy.<br>*Additional notes:* As evaluated by the physician because uniform and objective evaluation is not done across study groups. Other examples of instrumental ADL include cooking, cleaning, managing finances, and managing medications.
Paralytic, neuropathic, myopathic, and movement disorders	*Definition:* Paralytic, neuropathic (e.g., paresthesia, numbness, or pain), myopathic (e.g., generalized muscle weakness caused by rhabdomyolysis) or movement disorders (e.g., ataxia) emerging during or after anticancer therapy that substantially affects self-care ADL* for ≥12 months.*E.g., requiring daily assistance beyond what is considered age-appropriate with at least one self-care ADL and/or requiring instrumental aid, such as wheelchair or walking stick, for mobility.<br>*Time of ST:* Date when the condition fulfilling the clinical criteria in the ST definition has persisted for 12 months.<br>*Additional notes:* Examples of self-care ADL include grooming/personal hygiene, dressing, toileting/continence, transferring/ambulating, and eating.
Vocal cord paralysis	*Definition:* Persisting (≥12 months) vocal cord paralysis, either unilateral or bilateral, emerging during or after anticancer therapy, requiring ventilatory support (e.g., non-invasive ventilation) or tracheostomy, or leading to substantially reduced ability or inability to produce speech sounds.<br>*Time of ST:* Date when the condition fulfilling the clinical criteria in the ST definition has persisted for 12 months.
Cytopenia	*Definition:* Profound and permanent cytopenia in one or more hematopoietic cell lines, without evidence of hematopoietic recovery, emerging during or after anticancer therapy and requiring HSCT.<br>*Time of ST:* Date when the patient is referred for HSCT due to cytopenia.<br>*Additional notes:* Myelodysplastic syndromes are captured as second malignant neoplasms.
Immunodeficiency	*Definition:* Permanent immunodeficiency emerging during or after anticancer therapy and requiring HSCT.<br>*Time of ST:* Date when the patient is referred for HSCT due to immunodeficiency.<br>*Additional notes:* Severe leukopenia requiring HSCT is classed as cytopenia.
Second malignant neoplasms and benign central nervous system tumors	*Definition:* Second malignant neoplasms or benign central nervous system tumors emerging during or after anticancer therapy.<br>*Time of ST:* Date when malignant neoplasm or benign central nervous system tumor is diagnosed.<br>*Additional notes:* Non-melanoma skin cancers are not included.

ADL=activities of daily living, HSCT=hematopoietic stem cell transplantation, PEG=percutaneous endoscopic gastrostomy, STFS=severe toxicity-free survival

*Equating to more than class 3 as per the modified Ross Classification System for children aged 0-17.9 years

**Equating to class 3 or more as per the New York Heart Association Failure Scale for adults

†Kwan P, Arzimanoglou A, Berg AT, Brodie MJ, Allen Hauser W, Mathern G, et al. Definition of drug resistant epilepsy: consensus proposal by the ad hoc Task Force of the ILAE Commission on Therapeutic Strategies. Epilepsia. 2010 Jun;51(6):1069–77.

1.1.1. Minor clarifications

During initial data collection for the current study, the following clarifications were made:

1. Osteonecrosis: Classification required both imaging-verified deformation and substantial impairment in activities of daily living (ADL) to meet the generic criteria of objectivity and persisting severity. The original reference to the Ponte di Legno Toxicity Working Group grade 4 osteonecrosis was removed to avoid misinterpretation. (2)

2. Facial palsy (Amputation and physical deformation): It was clarified that complete facial palsy only qualifies as a Severe Toxicity (ST) if persisting for ≥12 months aligning with the criteria applied to other chronic and potential reversible conditions (and the generic criterion on persisting severity).

1.1.2. Application of the 12 months duration criterion

For conditions that may be fluctuating and potentially reversible, such as neuropathy and heart failure, a pre-defined duration of 12 months was required to fulfil the ST definition. For these conditions, the earliest possible date for an ST event is 12 months after the first documentation of the required severity level.

For cognitive dysfunction and psychiatric disorders, the condition must persist for ≥ 12 months *after* the completion of antileukemic treatment, as transient symptoms may occur due to treatment-related side effects. Consequently, patients cannot be classified with this ST prior to 12 months post-treatment. Furthermore, if symptoms emerge during the treatment phase, the precise onset cannot be determined from the analyses, as the event is only registered at the 12-month mark following treatment completion– irrespective of the actual time of symptoms emergence.

2. Data collection

2.1. Workflow

ST data were obtained by medical chart review with the following, systematic approach

1) Overview of the patient from recent follow-up visits, end-of-treatment summaries, and admission notes from time of diagnosis
2) Search words related to each ST were provided to 1) identify ST and 2) identify relevant pre-existing conditions
3) If an ST was identified, the date of occurrence was registered

In case of doubt regarding classifying a condition as a ST, each individual case was discussed in the research group to ensure consistency.

2.2. Supplementary classification procedures

<u>For conditions with 12-months duration criterion</u>

Due to the potentially progressing and/or fluctuating nature of certain conditions (e.g., neuropathy and osteonecrosis) combined with the fact that patients may not be hospitalized during the 12 months period, accurately determining the exact onset of fulfilling the severity criteria can be challenging. To enhance uniform classification, a standardized data collection procedure was applied specifically for conditions with 12-months duration requirement:

1. Identify the earliest date at which the condition fulfilled the predefined severity criteria.
2. Verify that the condition persisted for a minimum duration of 12 months.

3. Record both the initial date when severity criteria were first met, and the subsequent date at which it could be confirmed that the condition had been sustained for ≥ 12 months.
4. Determine the most recent timepoint prior to meeting the severity criteria was not yet classified with the condition
5. Determine whether the condition continued to meet the severity criteria at the time of last follow-up. If not, record the date on which the condition no longer fulfilled the ST definition. Notably, such conditions were still classified as ST, provided they had met the criteria for a minimum of 12 months, regardless of a subsequent resolution.

Multiple criteria for classification
Some ST could be classified in more than one way, such as either clinical severity or a proxy event such as joint arthroplasty (osteonecrosis) or organ transplantation (e.g., hepatic failure). In cases where both criteria were met, the earliest date was recorded as the ST event.

Transplantation (solid organ or bone marrow)
Patients referred to transplantation, but ultimately not transplanted due to clinically contraindications, were still classified with an ST– reflecting the severity of the underlying condition.

Onset during or after antileukemic treatment
All conditions fulfilling the criteria were classified as ST whether they occurred during or after treatment in accordance with the modified consensus definitions. (1)

Multiple Severe Toxicities
Some toxicities may occur more than once. This was possible to register for the ST: coronary artery disease, heart valve dysfunction, osteonecrosis, and SMN.

2.3. Pre-existing conditions

Relevant pre-existing conditions were identified for each type of ST for all patients. In cases where patients met the full ST criteria *prior to* their leukemia diagnosis, the condition was not classified as an ST event. Patients remained eligible for inclusion in analyses assessing the occurrence of any ST (e.g., STFS or cumulative incidence of ST), as they could still develop other types of ST during follow-up. For instance, a patient with pre-existing insulin-dependent diabetes prior to leukemia diagnosis remained at risk for other ST but was excluded from analyses exploring the cumulative incidence of insulin-dependent diabetes specifically. Consequently, no patients were excluded from the overall time-to-any-ST analysis based on pre-existing conditions alone.

Supplementary Table 2: Examples of relevant preexisting and predisposing conditions

**Severe Toxicity**	**Examples of pre-existing/pre-disposing condition**
Hearing loss	Hearing loss in one or both ears
Blindness	Impaired visual acuity in one or both eyes
Heart failure	Pre-existing cardiac dysfunction
Coronary artery disease	Familial hypercholesterolemia
Arrhythmia	Known predisposition to arrhythmia
Heart valve disease	Congenital valve disease
Gastrointestinal failure	Inflammatory bowel disease
Hepatic failure	Hepatitis virus carrier
Insulin dependent diabetes	Pre-existing diabetes
Renal failure	Pre-existing renal insufficiency
Pulmonary failure	Chronic lung disease
Osteonecrosis	Sickle cell disease
Amputation and physical deformation	Pre-existing scleroderma, previous amputation
Cognitive dysfunction	Autism, not having met normal developmental milestones, evidence of developmental delay at time of ALL diagnosis
Seizures	Pre-existing epilepsy
Psychiatric disease	Pre-existing psychiatric disorder
Paralytic, neuropathic, myopathic, and movement disorders	Charcot-Marie-Tooth
Vocal cord paralysis	Neurological disease
Cytopenia	Fanconi anemia
Immunodeficiency	Common variable immunodeficiency
Second malignant cancer and benign CNS tumor	Known cancer prone syndrome

2.4. Cognitive dysfunction

The following sections regarding classification of cognitive dysfunction was provided the data collectors to support the classification process.

Classification issues and challenges
The modified consensus definitions aim to guide the Severe Toxicity classification to be as uniform and objective as possible. Yet, the classification of 'Cognitive dysfunction' as a Severe Toxicity remains a challenge in some cases. In the following, we will address the challenges and issues associated with this classification.

Evaluation of instrumental ADL in children
The definition requires the cognitive dysfunction to be severe enough to result in "*severely restricted participation in school, vocational training, practice, and career, and/or requiring daily assistance beyond what is considered age-appropriate with other key activities of instrumental ADL".*

Examples of instrumental ADL include complex skills, such as cooking, cleaning, managing finances, and managing medications, that cannot be evaluated in children. The classification in children will therefore be based on the child's persistent requirement of assistance to manage daily activities (beyond requiring support in school) and their ability to participate in school (which should be "severely restricted" for classification as a Severe Toxicity).

Reasons for severely restricted participation in school
There may be many reasons for a child to not go to school and several factors often play a role including psychological, social, cultural, and parental factors. It is not possible to differentiate between the causes and often they will be overlapping. If the only issue is that the child has severely restricted participation in school, these cases will be graded as 'Possible cognitive dysfunction' given the other criteria in the definition (having substantial neurocognitive impairment and duration for ≥12 months after ending anticancer therapy) are fulfilled.

Neuropsychological testing
Several different neuropsychological test instruments exist and there is no international consensus on which to use and when. Furthermore, the patients with the most severe cases of cognitive dysfunction (e.g., severe brain damage) will not be able to complete neuropsychological teST. Therefore, neuropsychological testing is not required for the classification of 'Cognitive dysfunction' as a Severe Toxicity. If the neurocognitive impairment is documented with neuropsychological test results below normal range, the patient will only be classified with cognitive dysfunction if the other criteria are also fulfilled (substantially affected instrumental ADL and duration for ≥12 months after ending anticancer therapy).

Patients are not routinely tested neuropsychologically at the time of ALL diagnosis, hence no "baseline" is available and in most cases, the cognitive status of the child before ALL diagnosis will not be known. It should, however, be registered if the patient had developmental delay or other known, relevant conditions prior to the time of ALL diagnosis.

Duration criterion of ≥12 months after ending anticancer therapy

Anticancer therapy influences the patient's cognitive ability, and they are not expected to be able to participate fully in school during the treatment period. Therefore, patients can only be classified with 'Cognitive dysfunction' as a Severe Toxicity if the condition persist for ≥12 months after ending anticancer therapy. This requirement has the consequence that patients are "spared" from this event until reaching beyond this time point. This is intended (due to the expected influence of anticancer therapy on cognitive functioning) but may also lead to the exclusion of some cases that might seem obvious to classify based on the level of cognitive impairment (e.g., a patient with severe brain damage that dies or experiences a relapse before the duration criterion is fulfilled).

"Possible" and "Verified" cognitive dysfunction

Despite the efforts to support a uniform and objective registration, the classification of 'Cognitive dysfunction' as a Severe Toxicity is still (and will probably continue to be) associated with some degree of uncertainty. Therefore, this Severe Toxicity is graded into "Possible" and "Verified" cognitive dysfunction allowing future analyses to consider a worst and best-case scenario. The registration of 'Cognitive dysfunction' will therefore include an additional registration simply classifying the case as "Possible" or "Verified" cognitive dysfunction according to the definitions below. The data collector will be asked to (briefly) describe the reason for classifying the patient with this Severe Toxicity.

"Verified" cognitive dysfunction is defined as:

The patient lives in an institution due to cognitive dysfunction and/or the patient requires daily assistance with instrumental ADL due to cognitive dysfunction.*
*(*E.g. a child persistently in need of assistance to manage daily activities (beyond requiring support in school) or young adult where parents/other caretaker are in charge of instrumental ADL activities on behalf of the young adult.)*

"Possible" cognitive dysfunction is defined as:

The patient scores below normal range (<2.5 percentile) in neuropsychological test[§], but the degree of impact on instrumental ADL is uncertain and/or the patient has substantially impaired instrumental ADL, but it is uncertain if it depends on this specific condition or is due to other reasons, e.g., psychiatric disease.
§ Not specified due to variety in instruments used across centers. Neuropsychological testing is likely to be performed in patients with severe cognitive dysfunction but is not required for inclusion in the STFS measure.

Pre-existing conditions

Particularly for the youngest children (<1-2 years of age at time of ALL diagnosis), it can be difficult to determine if any neuropsychologically problems were present before the ALL diagnosis. It will likely not have been at focus at time of diagnosis and potential cognitive/developmental problems may not manifest until later in childhood. This issue will remain a limitation, but for children classified with cognitive dysfunction as a Severe Toxicity, we ask the data collector to explore if there were any indicators that the patient's cognitive problems were present (at some degree) already at the time of ALL diagnosis.

Example: “Verified” cognitive dysfunction

1. A 5-year-old child develops progressive leukoencephalopathy during antileukemic treatment resulting in persistent, severe brain damage. The patient lives at home with his parents but has no language and requires assistance with all daily activities.
2. A young man at 18 years of age finished antileukemic treatment 2 years ago. During treatment he developed septic shock with hypoxia. The patient is persistently described with cognitive dysfunction in several domains and his parents still take care of all instrumental ADL, such as managing finances, managing medication and doctor appointments etc. Before ALL diagnosis, the boy was described as normally developed and he went to high school without problems.

Example: “Possible” cognitive dysfunction

1. A 7-year-old child finished antileukemic treatment 2 years ago. The patient was normally developed before ALL diagnosis, but during the treatment, the child was described several times with difficulties of learning, concentration, and memory. Neuropsychological test were performed after ending antileukemic treatment and confirmed difficulties in several domains. 2 years after ending antileukemic treatment, the child still has severely restricted participation in school due to these problems.

3. Examples of Severe Toxicity cases

Supplementary Table 3. Examples of Severe Toxicity cases.

Severe Toxicity	Case examples
Blindness	Ex1: Neurological complication resulting in permanent blindness
Heart valve dysfunction	Ex1: Heart valve dysfunction requiring valve replacement, diagnosed years after treatment cessation
GI-failure	Ex1: PEG-tube due to neurological complications leading to physical inability to eat<br>Ex2: Stoma due to fungal sepsis with bowel involvement and persistent (>12 months) need for diverting stoma.
Hepatic failure	Ex1: Veno-occlusive disease during primary treatment leading to liver cirrhosis with repeated need for surgical interventions
Insulin dependent diabetes	Ex1: Insulin-dependent diabetes during treatment (including steroids)<br>Ex2: Pancreatitis during treatment; developed insulin dependent diabetes years later
Osteonecrosis	Ex1: Severe osteonecrosis occurring during treatment leading to significantly reduced mobility and requiring wheelchair for >12 months<br>Ex2: Osteonecrosis during treatment, years later resulting in joint arthroplasty
Amputation and physical deformation	Ex1: Amputation due to fungal sepsis
Cognitive dysfunction (verified)	Ex1: Anoxic brain damage following severe sepsis<br>Ex2: Severe, permanent neurotoxicity during relapse treatment
Seizures	Ex1: Neurological complications during treatment resulting in drug-resistant epilepsy
Psychiatric disease	Ex1: Several, severe psychiatric challenges in the years after ALL treatment. Functionally impaired to a degree that prevents participation in work, education, or independent management of daily activities.
Paralytic, neuropathic, myopathic, and movement disorders	Ex1: Vincristin-related incomplete paraplegia resulting in persistent need for wheelchair for mobilization.<br>Ex2: Severe central and peripheral neurological complications during relapse treatment resulting in persistent tetraplegia.<br>Ex3: Transverse myelitis during primary treatment; resulting in persistent paraplegia.
Second malignant neoplasms and benign central nervous system tumors	Ex1: Therapy-related myeloid neoplasm

4. Resolved Severe Toxicity cases

Several of the ST required a minimum duration of 12 months for classification. The 12 months persistence criterion was introduced in a previous Delphi process as an alternative to the term "permanent" which poses challenges when conducting statistical analyses.

The length of the timespan was based on two principles, as cited from Nielsen et al, 2023 (1):

"(1) When the condition has persisted for the specified duration, it is likely to be permanent (as specified in the original definition of hepatic failure), and/or

(2) when the condition has persisted for the specified duration, it is considered of such severity that it should be included as an ST even though it might improve over time to an extent by which it no longer fulfilled the clinical ST criteria (e.g., heart failure)."

In this study, 39 ST events were classified based on fulfilment of the 12-month criterion. To address potential limitations of this criterion, we investigated how many of these cases had resolved *after* meeting the severity criteria for ≥12 months.

Seven of the abovementioned 39 ST events had resolved at last day of follow-up (insulin-dependent diabetes (IDDM) and gastrointestinal failure (PEG-tube or stoma).

In addition, nine of the 39 ST events had improved at last day of follow-up. This included four cases of paralytic, neuropathic, myopathic, and movement disorders, three cases of osteonecrosis, and two cases of psychiatric disease. These instances do not indicate a full resolution of the condition, but rather reflect a degree of adaptation, where patients had learned to manage their disabilities over time.

The relevant ST are listed below in Supplementary Table 4 along with brief descriptions of the improved/resolved cases.

Supplementary Table 4. Short description of improved/resolved Severe Toxicities.

**Severe Toxicity**	**Number improved or resolved / total number**	**Description of improved/resolved cases**
Insulin-dependent diabetes	5/7	Five patients with insulin-dependent diabetes occurring during treatment and requiring insulin for ≥12 months. Insulin therapy was terminated after treatment cessation or after ending steroid treatment (in one case with graft versus host disease).<br><br>One of the patients remained on other antidiabetic medication.<br><br>One of the patients had developed pancreatitis during treatment.

**Severe Toxicity**	**Number improved or resolved / total number**	**Description of improved/resolved cases**
		One of the patients had previously had a diet-controlled diabetes.
Gastrointestinal failure	2/3	PEG-tube needed during treatment for ≥12 months due to physical inability to eat (severe mucositis). Removed later.<br><br>Diverting stoma during treatment ≥12 months. Removed later.
Paralytic, neuropathic, myopathic, and movement disorders	4/16	Neurological complications during primary treatment resulting in bilateral drop foot requiring instrumental aid for mobilization. Improves over the years with rehabilitation to a degree where the severity criterion is no longer fulfilled.<br><br>Neurological complications as a result of intracerebral hemorrhage. Longer period of neurorehabilitation. Improves over the years to a degree where the severity criterion is no longer fulfilled.<br><br>Two patients with leukemic infiltrations in the spine resulting in severely reduced mobility for ≥12 months. Improves over the years with rehabilitation to a degree where the severity criterion is no longer fulfilled.
Osteonecrosis	3/6*	One patient with severely reduced mobility for ≥12 months due to osteonecrosis; improved during bisphosphonate treatment.<br><br>Two patients with severely reduced mobility for ≥12 months due to osteonecrosis; spontaneous improvement over the years to a degree where the severity criterion is no longer fulfilled (yet still symptoms leading to repeated discussions of indication for joint arthroplasty)
Psychiatric disease	2/4	Two patients with several psychiatric challenges resulting in significant functionally impairment for ≥12 months; however, daily functioning improves over years with longer psychiatric treatment courses including medical treatment.
Hepatic failure	0/1	-
Cognitive dysfunction (verified)	0/2	-

*Unique patients with osteonecrosis classified as an ST by fulfilment of the 12-months-criterion. Some of these patients had involvement of multiple joints.

5. Analyses

5.1. Cumulative incidence of Severe Toxicities by risk groups

The cumulative incidence of Severe Toxicities was estimated using the Aalen-Johansen method treating death as competing risk. The cumulative incidence by risk group (standard risk = SR; intermediate risk = IR; high risk = HR) was explored in unadjusted analyses.

5.1.1. Risk group distribution across age categories

Older patients are more likely to be assigned to higher risk groups. Risk group distribution for this cohort is shown in Supplementary Table 5.

Supplementary Table 5. Risk group distribution across age categories used in the fitted Cox models. SR = standard risk; IR = intermediate risk, HR = high risk

	**SR**	**IR**	**HR**
1-4 years	105 (51.7%)	78 (38.4%)	20 (9.9%)
5-9 years	35 (32.7%)	45 (42.1%)	27 (25.2%)
10-17 years	25 (26.0%)	41 (42.7%)	30 (31.3%)
18-45 years	16 (16.2%)	44 (44.4%)	39 (39.4%)

5.1.2. Cumulative incidence of Severe Toxicities across risk groups

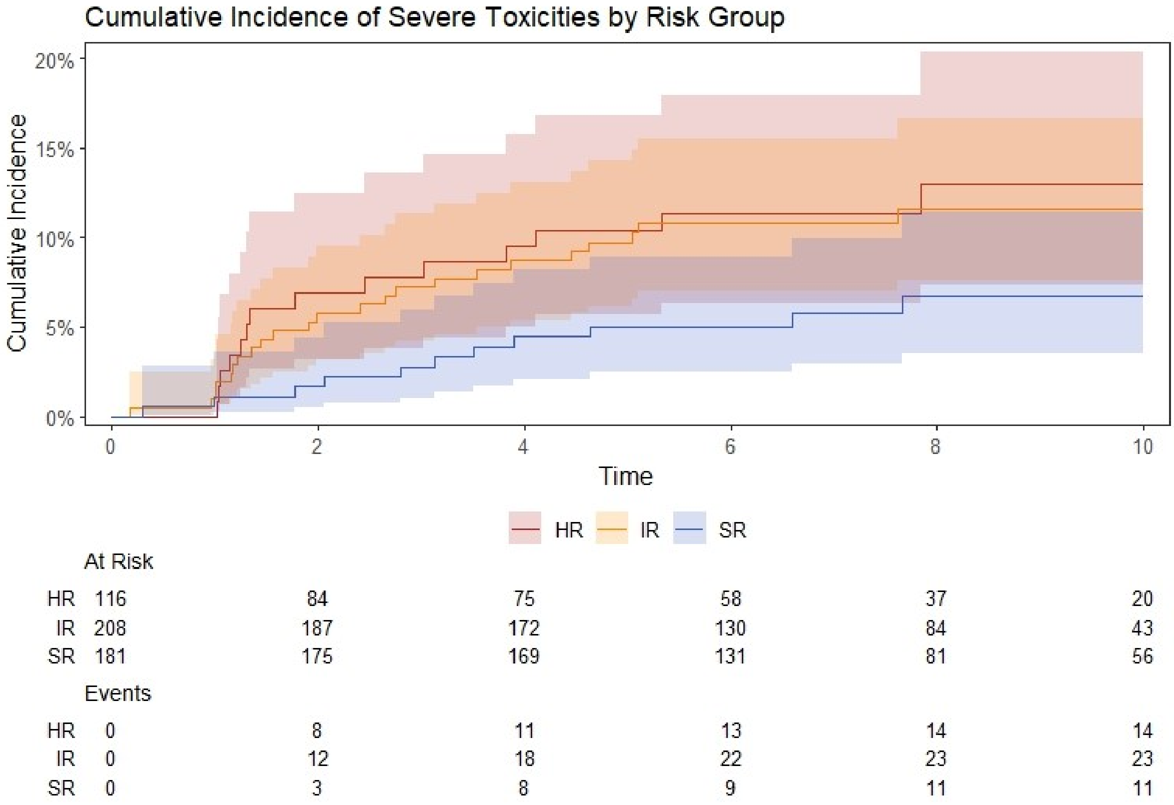

Supplementary Figure 1. *Cumulative incidence of Severe Toxicities by risk group (unadjusted).*

Supplementary Table 6. Cumulative incidence estimates (95% CI) by risk group

	**5 years**	**10 years**
SR	0.050 (0.025–0.089)	0.068 (0.035–0.115)
IR	0.097 (0.062–0.143)	0.116 (0.076–0.166)
HR	0.104 (0.057–0.168)	0.130 (0.073–0.204)

5.2. Cumulative incidence of Severe Toxicities (censoring at relapse)

Supplementary Figure 2 illustrates the cumulative incidence of Severe Toxicities when censoring at relapse with corresponding estimates.

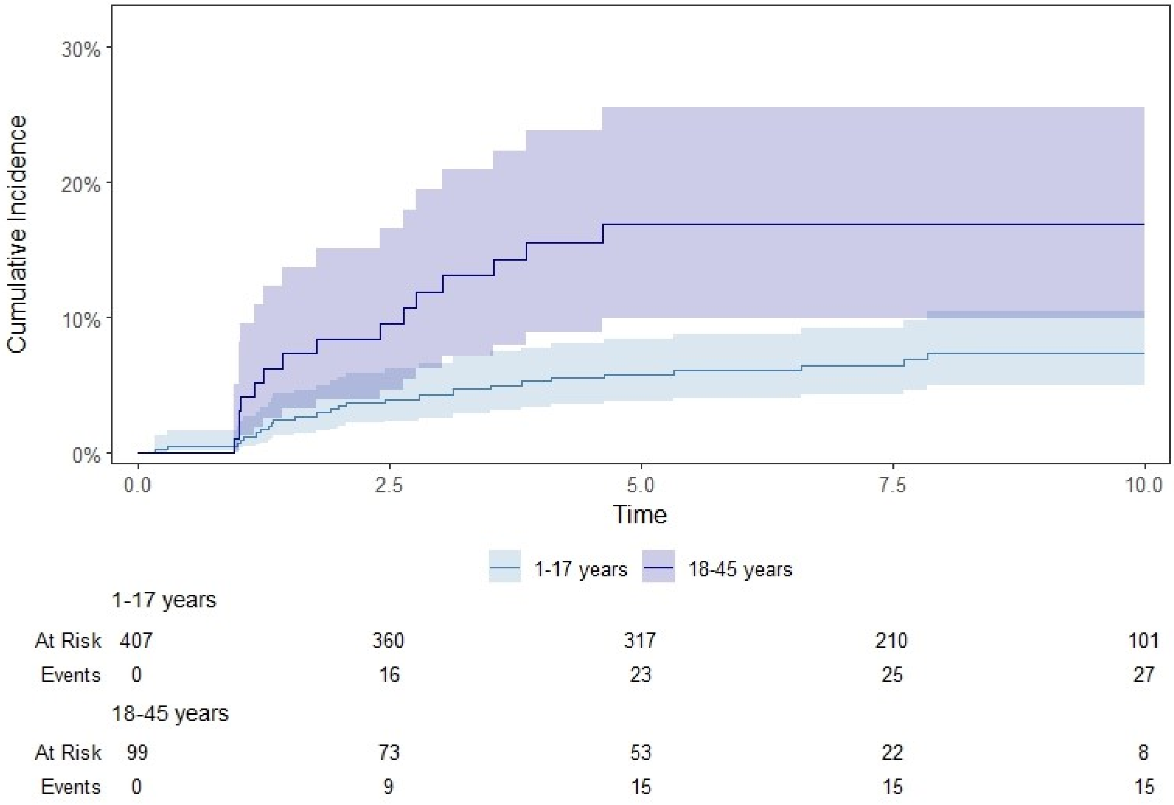

Supplementary Figure 2*. Cumulative incidence of Severe Toxicities among children and adults when censoring at relapse*

Supplementary Table 7. Cumulative incidence estimates (95% CI) at 5 and 10 years when censoring at relapse.

	**5 years**	**10 years**
1-17 years	0.058 (0.038–0.085)	0.074 (0.050–0.105)
18-45 years	0.169 (0.099–0.256)	0.169 (0.099–0.256)

5.3. Exploratory risk factor analyses

5.3.1. Cumulative incidence of Severe Toxicities by finer age groups

To explore age-related differences, the pediatric population was stratified into narrower age intervals: 1–4 years, 5–9 years, 10–17 years. Due to limited sample size in the adult population, it remained with the 18–45 years interval. Supplementary Figure 3 illustrates the cumulative incidence of ST across 4 age groups.

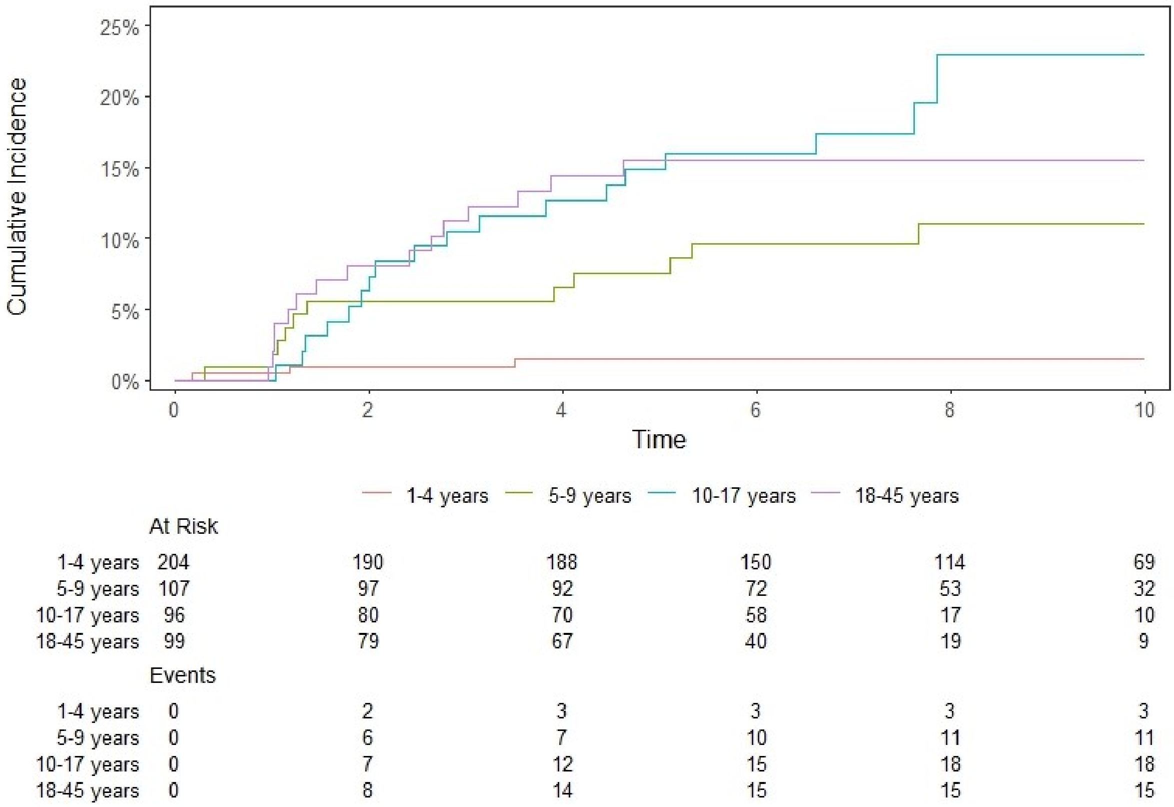

Supplementary Figure 3. *Cumulative incidence of Severe Toxicities in children aged 1–4 years, 5–9 years, and 10–17 years and in adults (18–45 years).*

Supplementary Table 8. Cumulative incidence estimates (95% CI) at 5 and 10 years.

	**5 years**	**10 years**
1–4 years	0.015 (0.004–0.040)	0.015 (0.004–0.040)
5–9 years	0.085 (0.042–0.148)	0.120 (0.065–0.194)
10–17 years	0.148 (0.085–0.228)	0.230 (0.133–0.343)
18–45 years	0.155 (0.091–0.235)	0.155 (0.091–0.235)

5.3.2. *Adjusted survival curves*

To visualize the predicted effect of age groups, we generated adjusted survival curves from a simplified Cox model, stratified by risk group and adjusted for sex. The time-dependent covariates relapse and HSCT were excluded, as they cannot be appropriately incorporated in predicted survival plots. Consequently, patients remained under observation following such events, which should be considered when interpreting these results. The curves are presented in Supplementary Figure 4 and are intended as illustrative, vizualising the isolated effect of age across risk groups.

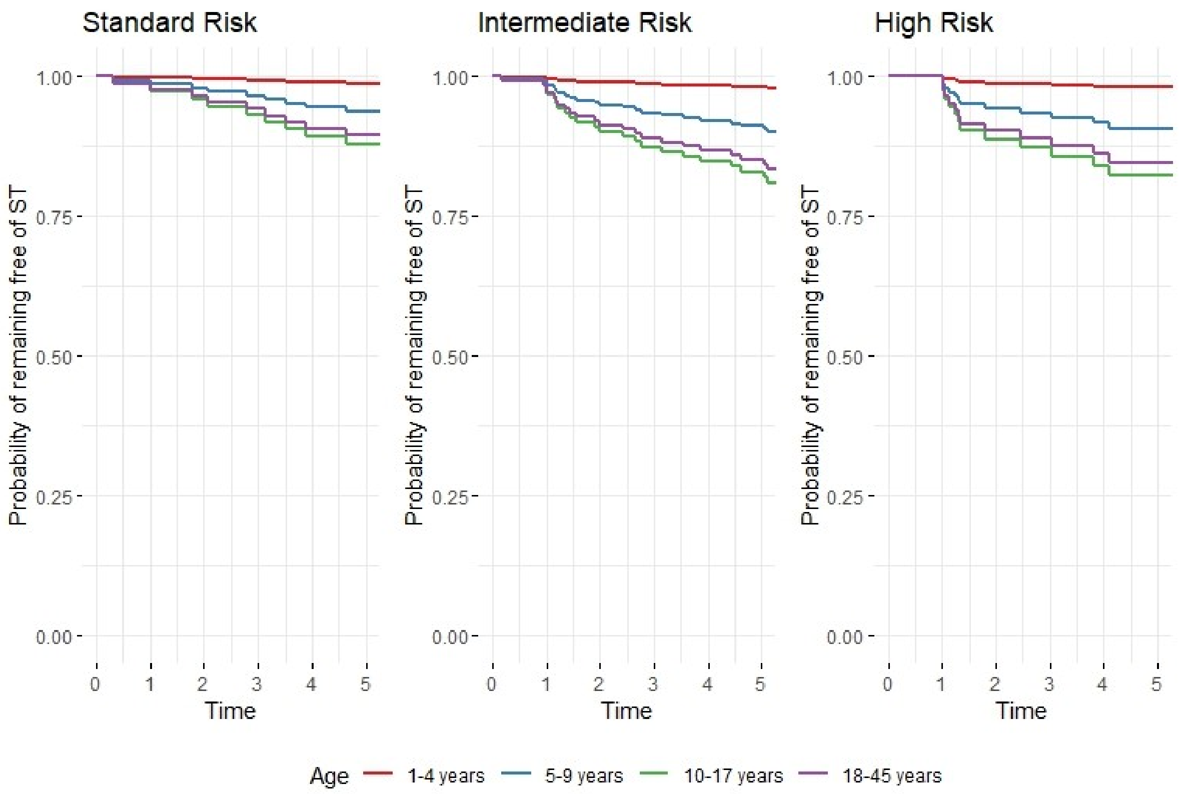

Supplementary Figure 4. *Survival curves showing the predicted effect of age group on the probability of remaining free of Severe Toxicities (ST) up to 5 years after diagnosis among patients alive and under observation. Based on a Cox model including age group and sex (fixed at male) as covariates, stratified by risk group. Time-dependent covariates (relapse and hematopoietic stem cell transplantation) were excluded, hereby demonstrating baseline risks.*

Supplementary Table 9. Predicted 5-year probabilities of remaining free of Severe Toxicities among patients who are alive. Estimates are based on a Cox proportional hazards model including sex and age group as covariates and stratified by risk group.

	**SR (95% CI)**	**IR (95% CI)**	**HR (95% CI)**
1–4 years	98.7 (97.4–100.0)	98.2 (96.3–100.0)	98.1 (96.0–100.0)
5–9 years	93.7 (88.6–99.2)	91.1 (84.9–97.7)	90.8 (83.9–98.3)
10–17 years	87.9 (79.6–97.0)	83.0 (74.3–92.8)	82.5 (72.1–94.3)
18–45 years	89.4 (81.2–98.5)	85.2 (76.5–94.7)	84.7 (75.5–95.2)

5.3.3. Cox proportional hazards models

We fitted a Cox proportional hazards model stratified by risk group to evaluate the association between age at diagnosis (categorized in four groups), gender, and relapse (included as a time-dependent covariate) on the hazard of developing Severe Toxicities (ST). The analysis included 505 unique patients (one excluded due to missing information about risk group) contributing a total of 607 time-intervals due to the use of relapse as a time-dependent covariate (tdc).

The model was stratified by risk group (SR, IR, HR) to account for baseline hazard differences across groups. Relapse was included as a time-dependent covariate, changing from 0 to 1 at the time of relapse, to appropriately model the effect of relapse on subsequent ST risk. The proportional hazards assumption was tested and met for all included covariates (global test p = 0.47). The model output is shown in Supplementary Table 5 and illustrated in Supplementary Figure 5.

Supplementary Table 10: Results from a Cox proportional hazards model assessing the association between age at diagnosis (categories), gender, and relapse (included as a time-dependent covariate) and the hazard of developing Severe Toxicities. The model was stratified by risk group to account for baseline hazard differences.

	**HR (95% CI)**	**p-value**
5-9 years	4.0 (1.4–11.3)	0.010
10-17 years	8.6 (3.1–24.0)	<0.001
18-45 years	6.6 (2.3–18.7)	<0.001
Sex (female)	1.4 (0.8–2.5)	0.28
Relapse (tdc)	6.4 (2.9–14.4)	<0.001

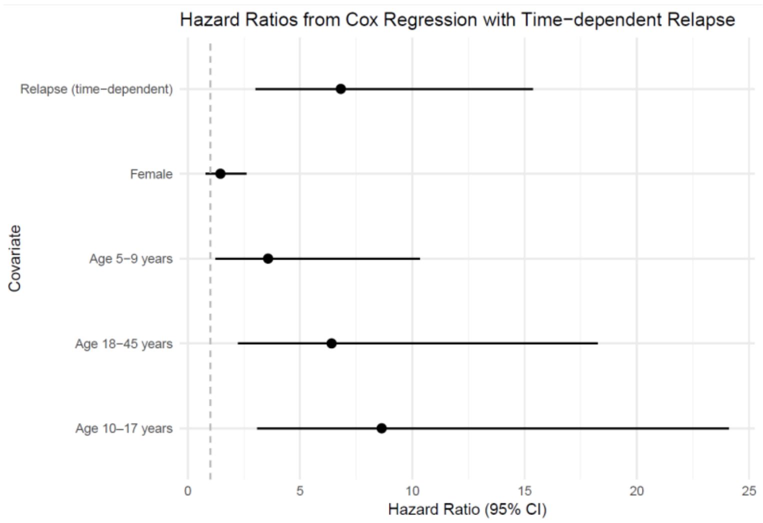

***Supplementary Figure 5**: Hazard ratio of ST risk.*

Interaction between age and risk group were assessed, but due to sparse events in some strata, the model did not converge, and interaction terms were therefore not included in the final model. Similarly, stratified Cox models within each risk group were attempted but were not feasible due to limited numbers of events.

<u>HSCT</u>

Additionally, we fitted a Cox model including hematopoietic stem cell transplantation (hSCT) as a time-dependent covariate (tdc) in addition to age and gender. The model was stratified by risk group to account for baseline hazard differences and patients were censored at time of relapse. The proportional hazards assumption was tested and met for all included covariates (global test p = 0.68). The model output is shown in Supplementary Table 11.

Supplementary Table 11. Results from a Cox proportional hazards model assessing the association between age at diagnosis (categories), gender, and hSCT (included as a time-dependent covariate) and the hazard of developing Severe Toxicities. The model was stratified by risk group and patients were censored at relapse.

	**HR (95% CI)**	**p-value**
5-9 years	4.8 (1.7–13.7)	0.003
10-17 years	9.1 (3.3–25.4)	<0.001
18-45 years	7.7 (2.7–22.1)	<0.001
Sex (female)	1.3 (0.8–2.2)	0.44
hSCT (tdc)	2.3 (0.9–5.9)	0.096

In the high-risk group we compared patients receiving hSCT in CR1 with those treated with chemotherapy alone. The Cox model was adjusted for sex and stratified by age. Patients were censored at relapse. The proportional hazards assumption was tested and met for all included covariates (global test p = 0.27). The model output is shown in Supplementary Table 12.

Supplementary Table 12. Results from a Cox proportional hazards model with hSCT as a time-dependent covariate, stratified by risk group and censored at relapse

	**HR (95% CI)**	**p-value**
hSCT (tdc)	2.0 (0.6–6.6)	0.256
Sex (female)	1.5 (0.5–4.5)	0.483

6. References

1. Nielsen CG, Thomsen BL, Als-Nielsen B, Conyers R, Jeha S, Mateos MK, et al. Physician-defined severe toxicities occurring during and after cancer treatment: Modified consensus definitions and clinical applicability in the evaluation of cancer treatment. Front Pediatr. 2023;26(11):1155449.

2. Schmiegelow K, Attarbaschi A, Barzilai S, Escherich G, Frandsen TL, Halsey C, et al. Consensus definitions of 14 severe acute toxic effects for childhood lymphoblastic leukaemia treatment: a Delphi consensus. Lancet Oncol. 2016;17(6):e231–9.
